# 微流控技术在外泌体分离分析中的研究进展

**DOI:** 10.3724/SP.J.1123.2021.07005

**Published:** 2021-09-08

**Authors:** Wenwen CHEN, Zhongqiao GAN, Jianhua QIN

**Affiliations:** 1.中国科学院大连化学物理研究所, 辽宁 大连 116023; 1. Dalian Institute of Chemical Physics, Chinese Academy of Sciences, Liaoning, 116023, China; 2.中国科学院大学, 北京 100049; 2. University of Chinese Academy of Sciences, Beijing 100049, China

**Keywords:** 外泌体, 微流控技术, 分离分析, exosomes, microfluidics, separation and analysis

## Abstract

外泌体是一类由细胞分泌的含有脂质、蛋白、核酸等多种物质的纳米级囊泡,主要参与细胞间的物质交换及信息传导,与多种疾病的发生发展密切相关。对外泌体进行深入研究,理解其生物学功能,对疾病诊断与治疗具有重要意义。由于外泌体尺寸较小且密度和体液接近,想要对复杂生物样本中的外泌体进行分离与分析十分困难。传统的外泌体分离方法如超速离心、超滤等大都需要借助大型仪器设备,且耗时长、操作复杂。因此迫切需要开发高效、便捷的外泌体分离检测手段。微流控技术因其微型化、高通量、可集成等特点,为外泌体的分离分析提供了一个新的平台。该文主要对近年来微流控技术在外泌体分离分析相关领域的研究进展进行了综述。重点从外泌体物理特性和生化特性两个角度出发,介绍了微流控芯片技术用于外泌体分离领域的主要原理、策略和方法。此外,还介绍了微流控技术与荧光、电化学传感、表面等离子体共振等多模态检测方法结合,实现外泌体一体化分析的新进展。最后,该文分析了目前微流控技术用于外泌体分离检测存在的挑战,并对其发展趋势和前景进行了展望。随着微流控外泌体分离分析装置的不断微型化、集成化、自动化,微流控芯片技术将在外泌体分离、生化检测、机制研究等方面将发挥越来越重要的作用。

## Abstract (en)

Exosomes are membrane-bound nanovesicles that are secreted by most types of cells and contain a range of biologically important molecules, including lipids, proteins, ribonucleic acids, etc. Emerging evidences show that exosomes can affect cells’ physiological status by transmitting molecular messages among cells. As such, exosomes are involved in various pathological processes. Studying exosomes is of great importance for understanding their biological functions and relevance to disease diagnosis. However, it is difficult to separate and analyze exosomes due to their small size, and because their density is similar to that of bodily fluids. Traditional methods, including ultracentrifugation and ultrafiltration are time-consuming and require expensive equipment. Other methods for exosome separation, including immunoaffinity-based methods, are expensive and rely heavily on specific antibodies. Precipitation-based methods do not yield acceptable purity for downstream analysis, due to polymer contamination. Thus, urgent demand exists for a portable, simple, affordable method for exosome separation. Microfluidic chip technology offers a potential platform for separation and detection of exosomes, with several remarkable characteristics, including low sample consumption, high throughput, and easy integration. This paper provides an overview of current microfluidic strategies for separation and analysis of circulating exosomes. In our introduction to exosome separation, we divide existing separation methods into two categories. Category one is based on exosome physical properties, and includes membrane filtration, nano-column array sorting, and physical isolation. The other is immune capture, which is based on biochemical characteristics of exosomes, and includes fixed base immune capture and unfixed base immune capture. In our introduction to exosome analyses, some commonly used methods, including western blotting, scanning electron microscopy, and flow cytometry are briefly described. Some new systems, which combine microfluidic technology with fluorescence, electrochemical sensing, surface plasmon resonance, or other multimodal analysis methods for integrated detection of exosomes are then described in detail. Finally, the challenges faced by microfluidic technology in improving exosome purity and making systems more portable are analyzed. Prospects for application of microfluidic chips in this area are also discussed. With the rapid development of micro/nano-manufacturing, new materials, and information technology, microfluidic exosome separation and analysis systems will become smaller, more integrated, and more automated. Microfluidic chip technology will play important roles in exosome separation, biochemical detection, and mechanism analysis.

外泌体是一类由细胞分泌的含有脂质、蛋白、核酸等多种物质的纳米级囊泡,其尺寸为30~150 nm^[1,2,3]^(见图1)。外泌体在人体中分布广泛,人体的外周血、尿液、乳汁、羊水等体液中均含有外泌体^[4,5]^。因外泌体可携带蛋白、运送RNA,其在人体中的角色主要包括物质的传递以及信息的传导两个大方面^[6,7]^。据此,人们将外泌体分为两种类型,第一种为有免疫活性的外泌体,其主要在抗原呈递和共刺激中发挥作用,第二种则是含有数量可观的RNA并可介导细胞间的遗传物质交流的外泌体^[8,9]^。外泌体与细胞的作用方式主要包括通过受体与靶细胞相互作用,激活下游细胞内的信号,以及直接与细胞膜融合从而将外泌体的膜蛋白整合到细胞浆膜或者是通过内吞作用将其转运分子传递至靶细胞的胞浆内。随着研究的深入,人们发现外泌体在适应性免疫^[10,11]^、炎症过程、胚胎形成^[12]^、肿瘤的发生与发展过程^[13,14]^中均发挥了重要的作用。以肿瘤为例,外泌体可以通过调节免疫功能,促进肿瘤血管新生以及肿瘤转移,或者直接作用于肿瘤细胞影响肿瘤进展^[15,16,17,18]^,因而外泌体获得了越来越多科学工作者的关注^[19]^。

**图1:**
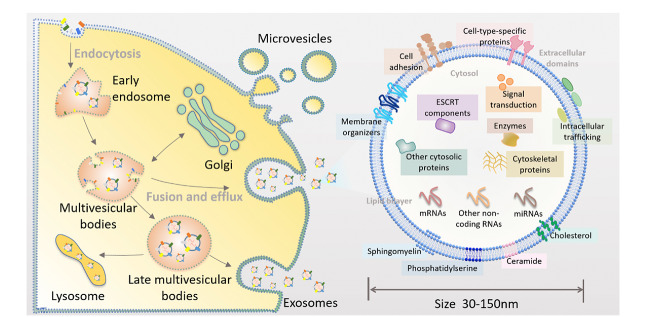
外泌体形成及组成示意图^[1]^

外泌体尺寸较小且其密度和体液接近,想要精准地对外泌体进行分离与检测十分困难。在过去的十几年中,微流控芯片技术因其具有微型化、高通量、可集成及低消耗等特点,为外泌体的精准分离分析提供了一种潜在平台。本文主要介绍了微流控芯片技术在外泌体分离分析相关研究领域的进展,并对其发展前景予以展望。

## 1 现有的外泌体分离方法

外泌体的高效分离与富集是对其研究的前提条件,目前较为常用的外泌体分离方法主要有超速离心、超滤、免疫亲和捕获、聚合物共沉淀以及基于微流控技术的外泌体分离方法等5种(见表1)。

**表1:** 不同外泌体分离方法的比较

Separation method	Principles	Sample volume	Sample	Advantages	Disadvantages
Ultra-centrifugation	size, density	large	cell culture medium, urine, et al.	without additional reagents	time consuming, instrument dependent, high shear stress
Ultrafilter	size	relatively large	cell culture medium, urine, et al.	without additional reagents	impurities with similar size, high shear stress
Immunocapture	antigen-antibody reaction	relatively small	urine, blood, et al.	high specificity	expensive, rely heavily on specific antibodies
Precipitation	protein-polymer reaction	large	cell culture medium, urine, et al.	cheap, easy to operate	polymer contamination, low recovery rate
Microfluidic chip	according to different design	small	blood, urine, precise samples	fixable, integrable	small separation volume, complex fabrication

### 1.1 超速离心法

超速离心^[20]^分离外泌体是通过离心力作用使不同密度、大小的物质分离开来。主要可以分为两类:一类为差速离心,一类为密度梯度离心。

差速离心即通过改变离心的速度,从而改变离心力的大小,使不同密度的物质分级分离。在分离外泌体时,便是通过不断提高离心机的转速,先将细胞及细胞碎片分离出来,最后再将尺寸较小的外泌体分离出来。

而密度梯度离心则是使用一种密度能形成梯度(在离心管中其密度从上到下连续增高)又不会使所分离的物质凝聚或失活的溶剂系统对样本进行分离的方法。离心后各物质颗粒能够按其各自的比重平衡在相应的统计密度中形成区带,较为常用的密度梯度溶剂是蔗糖。实验结果表明,当采用蔗糖为梯度溶剂时,样品中的外泌体将在1.13~1.19 g/mL的密度范围内富集^[21]^。采用超速离心法通常较为耗时,且对设备依赖程度较高,以离心力为驱动力还有可能对囊泡造成损害,导致外泌体的破碎与损失。

### 1.2 超滤法

超滤^[22]^即在一定的压力作用下,使待分离液体通过具有一定孔径的特制薄膜,尺寸小于薄膜孔径的物质将通过薄膜,而尺寸大于薄膜孔径的物质将被截留在薄膜上。外泌体的尺寸一般为40~100 nm,因而可以通过利用不同截留尺寸的超滤膜将外泌体从样品中分离出来。使用超滤法分离外泌体操作简单,但是超滤所使用的压力可能会导致囊泡的形变和破裂,且膜孔易堵塞,使用该分离方法最终结果可能导致蛋白污染较多。

### 1.3 免疫亲和捕获法

基于免疫亲和捕获分离外泌体,主要依据是外泌体表面含有其特异性标记物,如CD9、CD63等。在平面或磁珠上包被抗标记物的抗体,当外泌体经过此平面或磁珠时,便可以与抗体结合从而分离出来^[23,24]^。此外,不同细胞分泌的外泌体其表面含有的抗原存在差异,可通过选择不同的抗体实现对不同的外泌体的提取与分离^[25]^。使用免疫亲和捕获法来捕获外泌体,特异性高、操作简便且不影响外泌体形态的完整性,但是效率较低,抗体价格昂贵,存在活性和批次差异。

### 1.4 聚合物共沉淀法

共沉淀法分离外泌体是通过在待分离样品中加入其他溶剂,改变样品中物质的溶解度及分散特性,从而将外泌体从溶液中分离出来。目前报道的最常用的共沉淀剂是聚乙二醇(polyethylene glycol, PEG)^[26]^, PEG可与疏水性蛋白和脂质分子结合共沉淀,早期人们^[27]^将其应用于从血清等样品中提取病毒,现在也被用来提取外泌体,加入PEG后先将沉淀出的蛋白等物质分离出来,然后静置,利用各物质沉淀速率不同将其分离。使用PEG沉淀法操作简单,但纯度和回收率较低,且聚合物的除去较为困难,因此想要广泛应用仍需要不断改进技术条件。

## 2 基于微流控技术的外泌体分离方法

目前基于微流控技术的外泌体分离方法主要分为两大类。一类是基于外泌体物理性质如尺寸等的分离方法,另一类为基于外泌体生化特性如表面蛋白表达等的分离方法^[28]^。

### 2.1 基于外泌体物理特性的分离方法

针对外泌体物理特性进行分离的研究主要集中在尺寸差异上,外泌体的尺寸为30~150 nm,而细胞的尺寸一般在微米数量级,其他的细胞外囊泡如微囊泡以及凋亡小体的大小也和外泌体存在差异,因而可以利用它们尺寸的差异将外泌体从混合物中分离出来。和其他的分离方法相比,使用基于尺寸的方式分离出的外泌体大小更加均一。在微流控芯片中,基于外泌体尺寸进行分离主要有两大类。一类为使用纳米孔膜、纳米阵列、微过滤器等器件结构直接对样品进行过滤,将外泌体从中分离出来。另一类使用流场技术,通过施加动力作用或场作用使外泌体和其他物质分离开。此外,也可以将这两种方式结合进行外泌体的分离,在接下来的文章当中将对这两类分离方式进行详细介绍。

2.1.1 纳米孔膜、纳米阵列过滤

在最近的研究中,有学者^[29]^报道了一种使用双层膜过滤分离尿液中外泌体的体系(见图2a):该微流控芯片的核心部分是两个滤膜,作者将经过离心前处理的样品加入芯片中,样品先流经孔径为200 nm的滤膜,去除样品中体积较大的物质如细胞碎片和一些较大的囊泡等;之后样品又流经一个孔径为30 nm的滤膜,此时样品中尺寸小于200 nm大于30 nm的物质都将被截流下来。后期可以直接在此芯片中对截流下的外泌体进行染色等操作。相较于传统的超速离心方式,双层膜过滤耗时短,设备简单。该装置的外泌体捕获率为81.3%,特异性为90%。Woo等^[30]^也利用了双层膜过滤的方式对外泌体进行分离(见图2b),他们将驱动力改成了离心力,装置可以实现对血液中尺寸为20~600 nm的囊泡的富集,分离效率大于95%。此外,也有学者^[31]^利用膜过滤的方式,研究出了外泌体分级分离的芯片(exosome total isolation chip, ExoTIC);该芯片的构造和过滤器类似,通过改变中间夹膜的孔径,实现对不同尺寸物质的截留。而将具有不同滤膜孔径的芯片串联起来,便可以实现对外泌体的分级分离;分离后将滤器中间的滤膜取出,并将其上截留的外泌体溶解到溶剂中去,实现对不同尺寸外泌体的分离与富集。

**图2:**
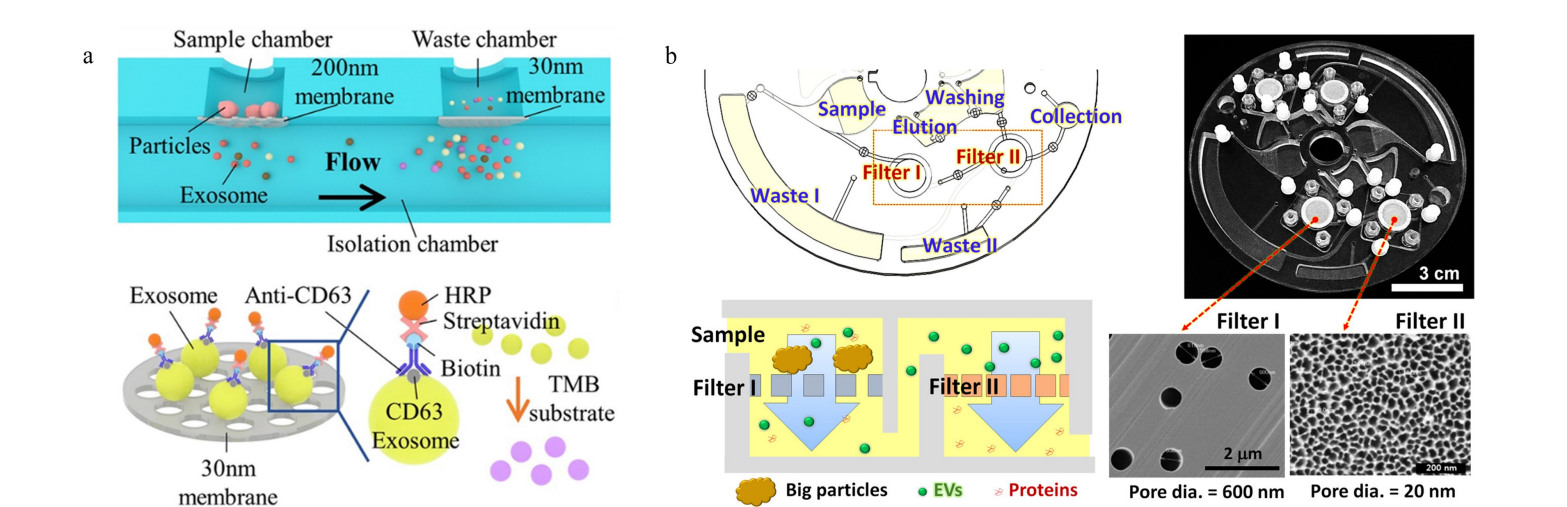
基于纳米孔膜、纳米阵列过滤分离外泌体的微流控方法

确定性侧向位移(deterministic lateral displacement, DLD)柱形微流控芯片是一种可以有效分离和富集微米级颗粒包括血液中的寄生虫、细菌、血细胞和循环肿瘤细胞等物质的技术。来自IBM、普林斯顿大学与西奈山伊坎医学院的科学家们合作设计并制造出了纳米级DLD(nano-DLD)芯片^[32]^,该芯片具有25~235 nm范围的均匀的间隙尺寸;经过实验他们发现,在低佩克莱数情况下,扩散和确定性位移产生竞争时,nano-DLD芯片仍然可以灵敏地将20~110 nm的颗粒分开。

无独有偶,来自美国纳米医学中心的研究者同样利用柱型微流控芯片实现了对外泌体的分离^[33]^。与前者不同的是,他们通过在形成的阵列微米柱上构建多孔硅的纤毛从而实现对外泌体的捕获与富集;通过控制合成条件,可以获得不同的纤毛形态及密度。当细胞、细胞碎片、外泌体以及蛋白等流经该带纤毛的微米柱阵列时,细胞以及细胞碎片因为体积过大,不能够被纤毛捕获,外泌体则可以被纤毛捕获,而蛋白等生物小分子因体积过小,会从纤毛的间隙溜走,因而此带有多孔硅纳米线的微柱阵列可以实现对外泌体的分离与富集。在实现捕获后可以通过加入磷酸盐缓冲液将多孔的纳米线结构溶解掉,从而将捕获到的外泌体完整地释放出来。

2.1.2 物理场分选

除了使用器件对样品进行直接过滤,人们也在微流控芯片上使用流场技术对外泌体进行分离^[34]^:杜克大学的学者利用声学技术和微流控芯片技术相结合(以下简称声波微流控),设计了一种可以不用标记及直接接触便可以实现外泌体分离和富集的方法。该声波微流控平台包含细胞去除以及外泌体分离两个模块。当施加一定频率的声波时,流体中的微粒将会受到声波辐射力的作用,其大小和粒子的体积成正比。而当微粒受到声波辐射力影响要发生位置的偏移时,又会受到一个斯托克斯拽力的作用,其大小与粒子的半径成正比。当粒子的体积增大10倍时,斯托克斯拽力增大10倍,而声波辐射力增大1000倍,因而体积越大的微粒,受声波场的影响就越明显,运动轨道偏移越大,利用此原理便可以将不同尺寸大小的粒子分离开来。使用该平台所获得的外泌体血液细胞的去除率达到99.999%。

将电场和磁场结合到微流控芯片上进行外泌体的分离近来也有报道。Cho等^[35]^提出了利用电场迁移来分离外泌体的方法。该方法在孔径为30 nm的渗透膜施加穿透膜的电场,在电场的作用下蛋白等发生迁移从而透过膜到外侧,而外泌体则会被截留在渗透膜上从而实现分离与富集。该方法分离效率较高,在尺寸上可以达到和超速离心类似的分离结果。和传统的超速离心方法相比,该种分离方式在RNA水平上的回收率为65%,比超速离心高了7.9倍,蛋白的去除效率在30 min内可以达到83.6%。

此外,也有学者^[36]^提出了一种利用黏弹流对外泌体进行分离的方法(见图3),该方法通过在样品中加入一种具有生物相容性的聚合物来控制作用在外泌体上的黏弹力从而实现对外泌体的分离,通过选择合适的浓度峰参数,最终所分离出的外泌体的纯度可大于90%,回收率大于80%。

**图3:**
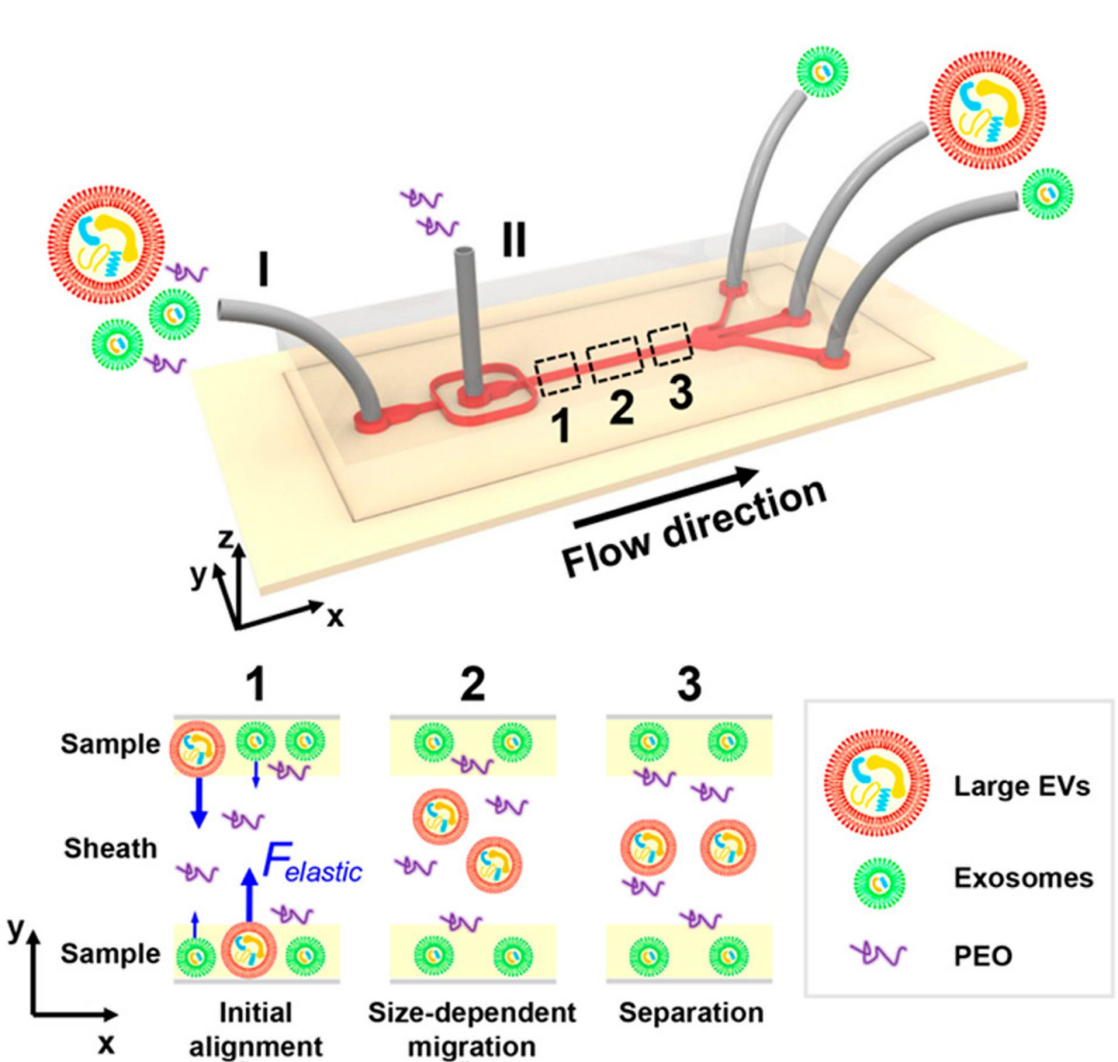
基于物理场分选分离外泌体的微流控方法

### 2.2 基于外泌体生化特性的分离方法

目前针对外泌体生化特性进行分离的研究主要集中在表面蛋白表达的差异上,通常通过免疫捕获的方式将外泌体从样本中分离出来。免疫捕获即将和外泌体相关的抗体吸附在固相载体表面,从而对外泌体进行捕获的技术。该技术具有快速、灵敏、简便、载体易于标准化等优点,因而被广泛使用。在微流控系统中采用免疫捕获的方式分离外泌体,从基底的状态上分可以分为固定基底免疫捕获和非固定基底免疫捕获两种。

2.2.1 固定基底免疫捕获

顾名思义,固定基底免疫捕获就是将捕获所需的抗体修饰在平面或有结构的基底上,但基底本身不可移动。较常用来作为标记物的蛋白是外泌体特异性的CD9、CD63以及CD81,此外针对一些具有自己特异性蛋白的外泌体,也可以用相应的蛋白抗体来进行捕获,如EpCAM^[37]^等蛋白。有学者^[38]^设计并制造了一种可以实时对外泌体进行定量分析的技术,该技术通过在平面底部修饰上外泌体特异性蛋白的抗体,实现对外泌体的捕获,接着通过表面等离子体成像(surface plasmon resonance imaging, SPRi)对信号进行分析。此外,将具有纳米结构的氧化石墨烯和聚多巴胺修饰在平面上,再修饰上捕获外泌体的抗体可以显著提高外泌体的捕获效率^[39]^(见图4a)。

**图4:**
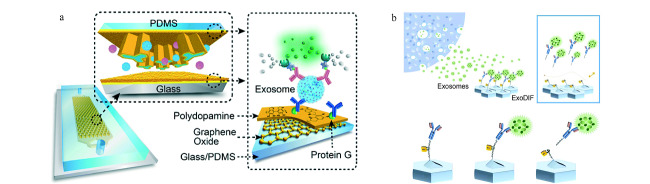
基于固定基底免疫捕获分离外泌体的微流控方法

Kang等^[40]^则提出了一种可以实现外泌体可逆捕获和释放的装置(见图4b)。该装置由两个不同的被免疫标记的具有结构的聚二甲基硅氧烷层组成,图案的设计结合机械旋转可以有效地提高抗体与外泌体结合的概率,从而可以在获得高的特异性的同时也获得高的外泌体的通量。作者在修饰过程中使用了具有双硫键的DTSSP(3,3'-二硫代双(磺酸琥珀酰亚氨基丙酸酯))这一物质,在捕获完成后,加入具有还原性的DTT(二硫苏糖醇)便可以成功地使双硫键断裂开,从而使所捕获到的外泌体被释放出来,以便于下游的进一步分析。

此外,人们也将一些微结构应用到了对外泌体的捕获上,如鱼骨结构^[41,42]^、三角结构等,这些微纳结构的加入,不仅可以增大捕获底面的比表面积,还可以增加液体的混合,提高捕获效率。Chen等^[43]^构建了一种纳米氧化锌包覆的三维支架芯片,三维支架相互连接的微孔使流体流动呈现混流或漩涡特征,氧化锌纳米线阵列为外泌体特异性抗体的固定提供了较大的表面积,纳米阵列具有尺寸排斥效应,对体积较大的囊泡有较好的去除作用。

2.2.2 非固定基底免疫捕获

和固定基底免疫捕获相对应,非固定基底免疫捕获即将捕获抗体修饰在可移动的基底上,微珠是目前较为常用的非固定外泌体捕获基底。外泌体的尺寸只有30~150 nm,直接分离的话较为困难,设备依赖性强。通过在微珠等表面修饰外泌体特异性的抗体等,便可以对外泌体进行捕获与富集。之后只要对捕获了外泌体的微珠进行操作便可以将外泌体分离出来,化小为大,操作将更为便捷。目前使用较多的微珠是磁珠和硅珠。Godwin等^[44]^将免疫捕获分离和外泌体的目标蛋白检测集合在微流控芯片上,开发了一种新的可以对外泌体蛋白直接进行分析检测的技术。该微流控芯片主要由两部分组成,第一部分为外泌体捕获单元,即加入修饰了抗体的磁珠,可以对外泌体进行捕获。第二部分为蛋白检测单元,加入蛋白提取剂,便可以在这个芯片上实现对外泌体蛋白的自动化检测。该实验装置可在100 min内实现对30 μL血液样品的检测。无独有偶,直接在芯片上实现对外泌体中的miRNA(micro-ribonucleic acid)检测的芯片也已有报道^[45]^,检测芯片由外泌体捕获、RNA捕获、反转录以及qPCR(quantitative real-time polymerase chain reaction)4个功能单元组成,外泌体在第一个腔室被表面标记有抗体的磁珠捕获后,加入RNA裂解液,裂解出的RNA进入到第二个腔室被捕获,之后洗脱出来,进入第三个腔室进行反转录,最后在第四个腔室进行qPCR,便可以对捕获的外泌体的RNA进行鉴定。此外,要对一种特定疾病的外泌体进行判定,有时需要同时对多个蛋白进行检测,这一点,也已经有学者利用免疫标记的磁珠实现^[46]^:研究人员以卵巢癌分泌的外泌体为例,用CD9的抗体来进行捕获,对CA-125、EpCAM、CD24这3种蛋白进行了免疫荧光标记,并对其光强等进行了检测,实验结果表明该方式可以实现对外泌体的多种标志蛋白的检测。

而使用不带磁性的微珠进行捕获,通常是在捕获之后利用滤膜截流以及通过流场的作用将吸附了外泌体的微珠分离出来。Dudani等^[47]^提出了一种可以将外泌体分离与外泌体荧光检测同步进行的技术(见图5a):作者使用了表面标记有CD63抗体的微球,将其和含有外泌体的样品以及表面标记有藻红蛋白的anti-CD81一起培养,之后再将微球和缓冲盐溶液一起从不同的入口注入微流控芯片中,在流体惯性力的作用下,微珠将迁移到缓冲液中,从而被分离出来;在微珠收集口处有个荧光检测器,可以实现对外泌体荧光的检测。

**图5:**
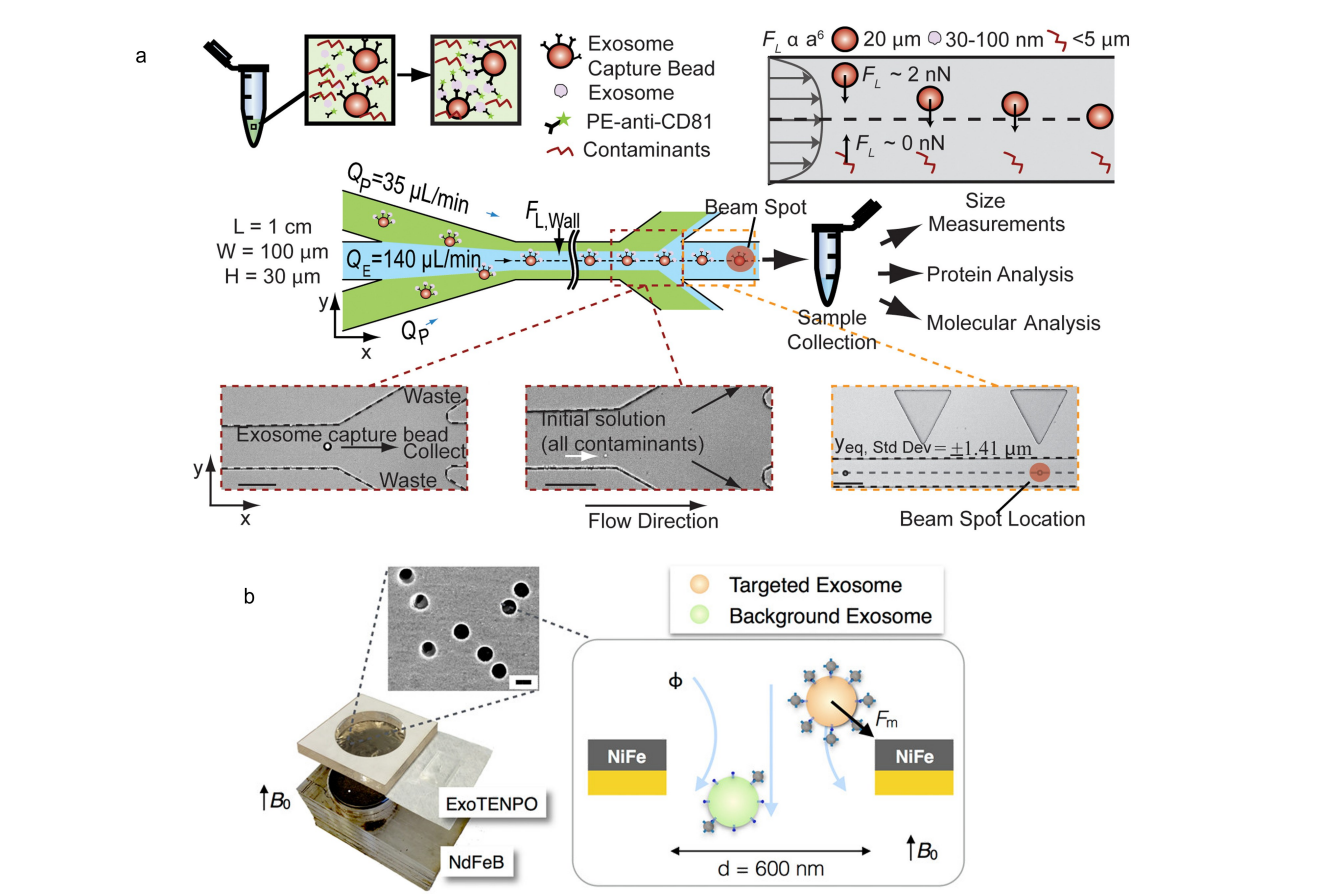
基于非固定基底免疫捕获分离外泌体的微流控方法

除了使用相对外泌体而言体积较大的微珠,比外泌体尺寸小很多的磁性纳米颗粒^[48]^也被应用于对外泌体的分离中(见图5b)。通过在磁性纳米颗粒表面修饰上相应的抗体,可以使其和外泌体特异性结合而使外泌体带有磁性,接着便可以通过对磁场的操控将外泌体从磁性纳米孔(exosome track-etched magnetic nanopore, ExoTENPO)中分离出来^[48]^。

表2对上述提到的方法进行了归纳与总结。

**表2:** 基于微流控技术的外泌体分离方法

Microfluidic technologies	Sample		Sample volume/μL	Recovery/ %	Time/ min	Isolated size/nm	Ref.
Based on physical characteristics of exosomes							
Membrane filtration							
Exodisc: double membranes		urine	1000	>95	30	20-600	[30]
ExoTIC: multi-membranes		culture media, plasma, urine	5000	>90	60	~30-100	[31]
Nano-column arrays							
Nano-DLD sorting		urine, serum	900	~50	60	~30-200	[32]
Ciliated micropillar array		liposomes	100	~60	10	~30-200	[33]
Physical field							
Acoustofluidic collection		human whole blood	500	99	50	~100	[34]
Electric field		mouse whole blood	1000	65	50	~10-400	[35]
Viscoelastic flow		fetal bovine serum	100	93.6	10	<200	[36]
Based on biochemical characteristics of exosomes							
Immune capture on fixed base							
SPRi antibody microarray		cell culture media	300	N/A	1	~70	[38]
Nano-IMEX		plasma	20	~80	40	<150	[39]
-COC^EV^HB-chip		plasma	1000	94	60	~100	[41]
ZnO chip		cell culture media, blood	100	>70	10	30-150	[43]
Immune capture on unfixed base							
ExoSearch chip: magnetic beads		plasma	1000	~79.7	10	<150	[46]
Polystyrene beads		cell culture media	700	N/A	10	60-90	[47]
ExoTENPO chip: magnetic nanoparticles		plasma	10000	N/A	60	~138-161	[48]

N/A: not applicable.

## 3 现有的外泌体分析方法

外泌体的分析一般是指对其形貌、粒径分布和携带的生化信息等参数的表征。围绕这些方面,现有的外泌体分析方法主要有显微镜图像观测、光散射粒径分析、可调电阻脉冲传感、抗体检测、流式细胞术等。

### 3.1 显微镜图像观测

外泌体的尺寸在100 nm左右,传统的光学显微镜因衍射极限的限制无法观察到这类囊泡^[49]^。近几十年,电子显微镜(扫描电子显微镜和透射电子显微镜)和原子力显微镜在纳米级别样品表征中快速兴起,逐渐成为外泌体研究的重要工具。

扫描电子显微镜(scanning electron microscope, SEM)利用电磁透镜将电子束在样品表面聚焦,通过高能电子束与样品相互作用产生二次电子、反射电子、X射线等信号,获得样品表面信息,具有景深大、放大范围广、制样简单等诸多优点,常用于外泌体形态和粒径的表征^[50]^。相比于SEM,透射电子显微镜(transmission Electron Microscope, TEM)具有更高的分辨率(低于1 nm)^[51,52]^,不足的是,由于TEM是在真空中进行的,生物材料需要固定和脱水,会影响外泌体的尺寸和形态,烦琐的样品制备过程也使测量时间以小时为单位^[53,54]^。

原子力显微镜(atomic force microscope, AFM)是一类通过分析样品表面的原子与微悬臂探针尖端的原子间的相互作用力来研究物质的表面结构及性质的扫描探针显微镜,其分辨率可达亚纳米级^[55,56,57]^。由于原子力显微镜极高的分辨率,外泌体必须结合在一个非常平坦的表面上,例如云母,而抗体可以用来将外泌体与表面结合,这样也可以获得生化信息。由于使用抗体与表面结合的微囊效率未知,因此微泡的浓度无法确定。此外,表面结合可能会影响微泡的形态,这可能会阻碍真实直径的测定^[53]^。

### 3.2 光散射粒径分析

在外泌体的分析检测中,光散射技术通常用于表征外泌体的尺寸分布。

小角X射线散射(small angle X-ray scattering, SAXS)是一项基于X射线光子在低角度下对样品电子弹性散射的分析技术,可在1~200 nm范围提供样品结构信息^[58,59]^。值得注意的是,SAXS在对样品粒径进行表征时,需要单分散、高浓度的样品^[58]^。

动态光散射(dynamic light scattering, DLS)是一项测定胶体悬浮液粒径的常用技术^[60]^。其原理在于将布朗运动与粒子的大小联系起来,利用这种技术可以检测直径在1 nm到6 μm之间的粒径分布^[61]^,是外泌体粒径表征的常用方法^[62,63]^。但其受水化层等界面因素的影响,测得的尺寸要比外泌体真实尺寸偏大^[54]^。

纳米颗粒跟踪分析(nanoparticle tracking analysis, NTA)是一种可以对外泌体的尺寸和浓度进行表征的技术。在NTA方法中,主要通过跟踪单个外泌体随时间的移动并计算每外泌体的均方位移,确定流体动力学半径并显示出外泌体的大小分布^[64,65]^。

### 3.3 可调电阻脉冲传感

可调电阻脉冲传感(tunable resistance pulse sensor, TRPS)是一项单粒子原位表征技术^[66]^,该检测系统主要由带有两个电极的流体腔室和一块弹性绝缘聚氨酯膜组成,在聚氨酯膜中含有一个圆锥形状的纳米孔。通过监测粒子通过纳米孔时产生的脉冲信号,可以精确测量溶液中外泌体的浓度和粒径分布。相比DLS, NTA等技术,TRPS不依赖布朗运动,样品用量少,测量速度快,可同时对外泌体的尺寸、浓度和zeta电位等重要参数进行测定^[67,68]^。

### 3.4 抗体检测

抗体检测主要依据抗体和抗原的相互作用,将外泌体表面的蛋白表达转化为荧光、吸光等可测量信号从而对外泌体进行分析。目前在外泌体蛋白检测中最常用的是蛋白印迹法(western blot)该方法具有特异性强、易用性高等特点^[69]^,可根据CD81、HSP70等蛋白的存在情况辅助外泌体的鉴定^[70]^。酶联免疫吸附测定(enzyme linked immunosorbent assay, ELISA)也是一种常用来对外泌体蛋白进行检测的方法^[71]^,相比于Western blot, ELISA在蛋白的定量分析中精度更高,但依赖于酶标仪等设备,且不能得到蛋白相对分子质量等信息。此外,以生成有色化合物的显色反应为基础的比色分析法在外泌体蛋白的检测中也有一定的应用,该方法可以在几分钟内对外泌体表面蛋白的细微差异进行表征^[72]^

### 3.5 流式细胞术

流式细胞术是一种集细胞高通量分选和多参数检测于一体的精密技术^[73]^,流式细胞仪在细胞研究中的成功运用使得其在外泌体检测上也开始受到广泛关注^[74]^。贝克曼等一些公司建立的纳米流式细胞仪(nano-flow cytometer, nano-FCM)能够检测到尺寸小于100 nm的外泌体相关囊泡,随着流式技术的改进和外泌体特异性标记试剂的开发,流式细胞术有望在该领域发挥举足轻重的作用^[56]^。

接下来,在表3中对上述提到的外泌体常用检测方法进行了归纳与总结。

**表3:** 不同外泌体分析方法的比较

Analysis method	Principles	Analysis objects	Ref.
Microscopy	the reaction between samples and electrons or detection	size, morphology	[50-52,55-57]
	probes		
Light scattering	change of light scattering intensity	size distribution	[54,58,62,63,65]
TRPS	change of conductivity	size, concentration, zeta potential	[67,68]
Antibody detection	antigen-antibody reaction	proteins	[69,71,72]
Nano-FCM	the scattering light and fluorescent light of detected cells	size, biochemical characterization	[73,74]

## 4 基于微流控技术的外泌体分析方法

随着技术的进步,外泌体检测的各种方法不断涌现,前面介绍的方法往往需要相对庞大和复杂的实验室基础设施。近年来,微流控技术高速发展,许多基于微流控技术的外泌体检测方法不断被报道,这些检测方法具有分析速度快、灵敏度高、试剂消耗少、携带方便等优点。此外,微流控技术易于模块化并可多单元集成,这意味着可以将外泌体的纯化、富集和检测整合到微流控平台上,为临床发现外泌体生物标志物和对肿瘤等疾病的诊断与预后提供巨大的潜力^[75]^。目前基于微流控的外泌体分析方法大致可以分为:荧光检测、表面等离子体共振、核磁共振、数字液滴PCR、电化学传感等,下面将对提到的这几种方法进行详细的介绍。

### 4.1 荧光检测

荧光是一种物质在吸收光照或者其他电磁辐射后出现冷发光的现象。其荧光强度、波长、发光时间与材料的性质有关。因此,观测荧光的发射参数可以用来检测目标样品。荧光成像技术具有精度高、灵敏度高等优点,已广泛应用于微流控芯片的外泌体检测系统中。近期的研究报道中,Ai等^[50]^建立了一种微流控水动力分析平台,该平台首先利用微珠对溶液中的外泌体进行初步的捕获,然后利用微流控芯片将富集有外泌体的微珠进行分散与固定,在芯片上实现了外泌体的大规模多重荧光分析(见图6a)。

**图6:**
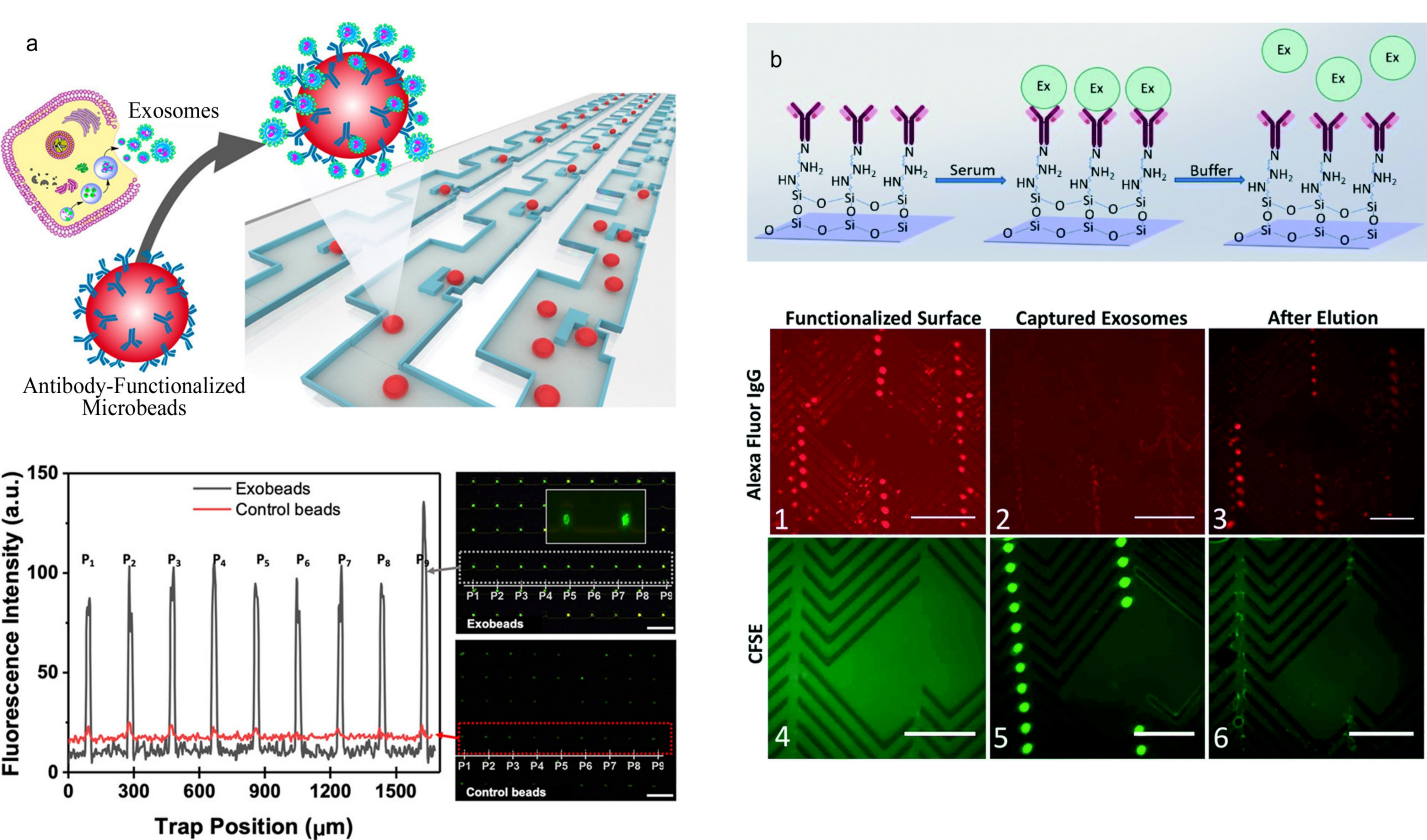
微流控芯片与荧光检测联用用于外泌体检测

而Hansford等^[76]^则构建了一种经过抗体功能化的人字形沟槽微流控装置,在实验中,研究人员将其用于高级别浆液性卵巢癌病人血清中外泌体的分析,利用荧光显微镜对微流控芯片捕获和释放的外泌体进行表征,从而实现了外泌体的洗脱过程的动态监测(见图6b)。流式细胞术(FCM)和微流控技术相结合在外泌体检测方面也有很大的潜力。一种基于微流控技术的多功能流式细胞仪成像检测系统已被用于卵巢癌的早期检测,该方法通过建立由平行纳米通道组成的流式细胞术阵列,成功地实现了外泌体的计数^[77]^。从发展趋势来看,基于微流控芯片的荧光检测方法在外泌体检测中的应用将会越来越广泛。

### 4.2 表面等离子体共振

表面等离子体共振(SPR)是一种无标签的实时跟踪传感技术,通过分子吸附在金属表面引起的折射率改变实现对生物分子的痕量分析。近年来,基于SPR技术的纳米传感器因其在分子检测上的高灵敏性而备受关注。Hu等^[78]^开发了一种基于SPR的外泌体微流控芯片传感器,实验中该团队将抗体修饰在金的表面,在利用抗体微阵列捕获外泌体后,金表面的折射率发生改变,实现了对肿瘤细胞培养液中的外泌体的定量检测,为外泌体的分离分析提供了一种简便、高效的策略。

### 4.3 核磁共振

核磁共振现象(nuclear magnetic resonance, NMR)是一种基于磁矩不为零的原子核在外磁场作用下自旋能级发生塞曼分裂,共振吸收某一定频率的射频辐射的物理过程,由Purcell和Bloch在1946年发现^[79]^。NMR是化学分析中的一项重要技术,Lee等^[80]^将用于NMR测量的微线圈嵌入到微流控芯片的中间层,用抗体和外泌体作用后与磁性纳米颗粒进行偶联,使用内置微线圈进行核磁共振测量,以确定样品的R2值,实现了对外泌体膜蛋白的检测。

### 4.4 数字聚合酶链式反应

数字聚合酶链反应(dPCR)是一种绝对定量的方法,它将含有核酸模板的标准PCR反应体系,平均分配成上万个和数百万个PCR反应,使每个反应中尽可能含有一个模板分子,在单分子水平上进行大规模的平行PCR分析^[81]^。dPCR检测目标基因更可靠,不需要校准曲线。由于其高灵敏度,dPCR已广泛应用于多个领域,包括单细胞分析、突变检测、拷贝数变异分析、细菌和病毒检测等。可以设想,随着微尺度样品处理与dPCR分析的集成,该技术将成为临床样本中外泌体基因分析的有力工具^[82]^。

### 4.5 电化学传感

电化学分析方法因其具有成本低、时效性好、灵敏度高、样品量少等优点在生化检测领域受到广泛关注。在电化学检测过程中,将识别元件(如抗体、适配体等)修饰到化学电极上与外泌体特异性结合,再利用不同的电化学检测技术(包括伏安法、安培法、阻抗法、电位法等)对电极进行检测便可以获取样品中外泌体的浓度和表面蛋白等信息。Revzin等^[83]^报道了一种基于适配体的电化学生物传感器用于外泌体的定量检测:将外泌体跨膜蛋白CD63适配体固定在金电极表面上与含有亚甲基蓝标记的探针链杂交,当检测到外泌体时,探针链会释放出来,电化学信号以与分析物浓度成正比的方式降低;将该装置整合到微流控芯片中,可以对外泌体浓度在1×10^6^~1×10^8^个粒子/mL的样本进行定量检测。Ye等^[84]^则以聚二甲基硅氧烷为主要材料,构建了内部含有人字形微阵列结构的电化学检测芯片(见图7),当检测到外泌体跨膜蛋白CD63时,吸附有外泌体的磁珠可与一种DNA发卡结构结合,在血清素的作用下,发卡结构异构成G-四聚体结构,对过氧化氢分解具有高效的催化作用,而产生强烈的电化学信号,从而实现对外泌体的检测;在对临床血清样品中CD63阳性外泌体定量检测中,文中开发的两步级联微流控平台,可以实现对健康组和疾病组的外泌体含量的灵敏区分。

**图7:**
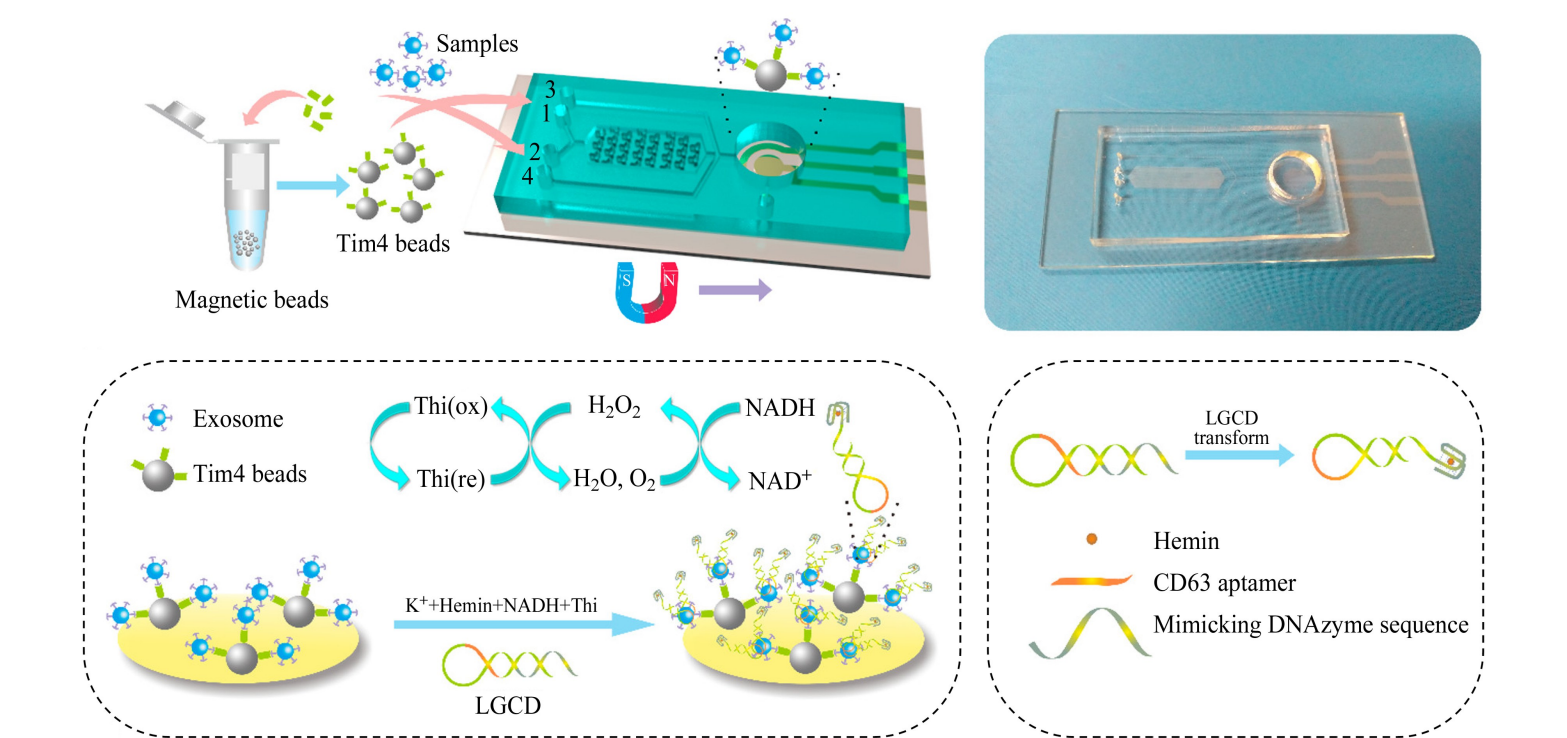
电化学传感检测外泌体

## 5 总结与展望

本文主要对现有的外泌体分离分析方法进行了综述,重点介绍了微流控技术在外泌体分离分析领域的研究进展。相较于传统的超速离心、超滤、免疫捕获及共沉淀,微流控技术体系更小,更加灵活,微流控免疫亲和方法可以分离出高纯度的外泌体,特异性强;基于外泌体物理特性的分离方法不需加入抗原抗体等昂贵试剂,成本低,分离过程不会引起外泌体表型变化,利于下游分析;基于微流控技术的外泌体分析方法分析速度快、通量高、试剂消耗少,可满足大量临床样本中外泌体的快速检测需求。因此,微流控技术在针对临床少量样本的外泌体分离及疾病快速检测等方面具有显著优势,为外泌体分离与分析提供了一个新的平台。

尽管微流控技术在外泌体分离分析领域已经取得了显著进展,但是目前通常需要和一些仪器如注射泵、荧光检测仪等进行联用,这在一定程度上限制了其应用场景。此外,因为外泌体尺寸小且密度和体液相近,单一的分离方法存在不足。如何平衡微流控芯片上外泌体纯度和回收率的关系,也面临很大的挑战。近年来,随着微纳制造、新材料、信息技术的快速发展,基于微流控技术的外泌体分离分析芯片的设计和配套装置性能将会获得进一步提升,主要体现在:(1)精密制造工艺的发展使在一块芯片上集成多种外泌体分离手段及实现外泌体分离检测一体化成为可能;(2)将芯片和便携式检测设备结合,构建微型化的外泌体微流控分离分析平台,实现外泌体的快速检测,大大拓展了其应用空间。随着微流控外泌体分离分析装置的微型化、集成化、自动化,微流控芯片技术将在外泌体分离、生化检测、机制研究等方面将发挥越来越重要的作用。
